# Retraction Note: The long noncoding RNA CRYBG3 induces aneuploidy by interfering with spindle assembly checkpoint via direct binding with Bub3

**DOI:** 10.1038/s41388-024-02997-3

**Published:** 2024-03-05

**Authors:** Ziyang Guo, Yingchu Dai, Wentao Hu, Yongsheng Zhang, Zhifei Cao, Weiwei Pei, Ningang Liu, Jing Nie, Anqing Wu, Weidong Mao, Lei Chang, Bingyan Li, Hailong Pei, Tom K. Hei, Guangming Zhou

**Affiliations:** 1 State Key Laboratory of Radiation Medicine and Protection, School of Radiation Medicine and Protection, Institute of Space Life Sciences, Medical College of Soochow University, Collaborative Innovation Center of Radiological Medicine of Jiangsu Higher Education Institutions, Suzhou, 215123 China; 2 Center for Radiological Research, College of Physician and Surgeons, Columbia University Medical Center, New York, NY USA; 3 Department of Pathology, the Second Affiliated Hospital, Medical College of Soochow University, Suzhou, 215123 China

Retraction to: *Oncogene* 10.1038/s41388-020-01601-8, published online 09 February 2021

The authors have retracted this article because of concerns about images in two of the figures.

In particular:

Figure 2G (panel Ctrl vs. Ctrl) appears to partially overlap with Figure 2G (panel Ctrl vs. NC)

Figure 2G (panel Ctrl vs. NC) appears to partially overlap with Figure 2G (panel sh1 vs. NC)

Figure 2G (panel sh2 vs. Ctrl) appears to partially overlap with Figure 2G (panel sh2 vs. NC)

The mice shown in Figure 5H (panel Ctrl) appear to be the same as the mice shown in Figure 5H (panel LNC CRYBG3+sh Bub3)

The authors were not able to access the original raw data for the study for verification and as a result, the authors no longer have confidence in the veracity of the data in this article.

All authors agree to this retraction.

